# Epidemiologic, Racial and Healthographic Mapping of Delaware Pediatric Cancer: 2004–2014

**DOI:** 10.3390/ijerph13010049

**Published:** 2015-12-22

**Authors:** Laurens Holmes, Jonathan Vandenberg, Lavisha McClarin, Kirk Dabney

**Affiliations:** 1 Nemours/Alfred I. duPont Hospital for Children, Office of Health Equity and Inclusion, 2200 Concord Pike, Wilmington, DE 19803, USA; jonathan.vandenberg@furman.edu (J.V.); lavisha.mcclarin@nemours.org (L.M.); kirk.dabney@nemours.org (K.D.); 2 Biological Sciences Department, University of Delaware, 118 Wolf Hall, Newark, DE 19716, USA; 3 Biological Sciences Department, Furman University, 3300 Poinsett Hwy, Greenville, SC 29613, USA; 4 Epidemiology & Biostatistics Department, University of Maryland-College Park, 255 Campus Drive, College Park, MD 20740, USA

**Keywords:** pediatric cancer, health disparities, cumulative incidence, Delaware

## Abstract

Childhood cancer remains the leading cause of disease-related death among children 0 to 14 years and incidence varies by race, ethnicity, sex, geographic locale, and age at onset. However, data are unavailable in some regions, indicative of a need for such information for cancer awareness, education and prevention program. We utilized retrospective epidemiologic design to assess and characterize pediatric tumors in the Nemours Electronic Medical Records, between 2004 and 2014. Tumor frequency and children population size were used to determine the period prevalence as cumulative incidence (CI) proportion, as well as chi-square and Poisson Regression. The CI for overall childhood cancer in Delaware was 234 per 100,000 children, and varied by race, black (273 per 100,000), white (189 per 100,000). Similarly, sex variability was observed in CI, boys (237 per 100,000) and girls (230 per 100,000). The most commonly diagnosed malignancies were acute lymphoblastic leukemia (ALL), Central Nervous System (CNS)/brain and renal cancer. The geographic locales with relatively higher cancer CI in the state of DE were zip codes 19804 and 19960, but this does not imply cancer clustering. Differences in overall childhood cancer distribution occurred by race, sex, geography, and age. These findings are indicative of the need for cancer-specific health education, awareness and prevention programs in reducing the observed disparities in Delaware.

## 1. Introduction

Cancer is the leading cause of disease-related death among children 0 to 14 years old [1]. In 2015, it was estimated that 10,380 new cases of pediatric cancer in children 0 to 14 years old would be diagnosed, with an estimated 1250 deaths [2]. Despite the estimated incidence and mortality, incidence rate for pediatric cancer has been approximately stable with a slight increase over the past two decades [3]. In 2004, for example, the incidence rate for invasive pediatric cancer in the United States for children 0 to 19 years old was approximately 17 per 100,000 children [4]. In 2014, ten years later, 15,780 new cases of pediatric cancer were expected to be diagnosed in children ages 0 to 19 [5]. Incidence rates, however, differed depending on the type of malignancy, with increasing rates observed in acute lymphoblastic leukemia (ALL), neuroblastoma, retinoblastoma, renal cancer, thyroid carcinoma, and CNS/brain tumors [5,6,7,8,9,10,11,12,13]. In addition, health disparities had been observed in incidence and survival [14,15].

Race, ethnicity, sex, age group, socioeconomic status, and geographic locale are common indicators of disparities in childhood cancer [16]. Racial disparities have been particularly evident in ALL, the most common childhood cancer, with higher incidence rates higher among white children despite poorer survival among black children [8,12]. Additionally, disparities have been observed in incidence by sex, specifically in pediatric leukemia and thyroid cancer [8,9]. Incidence rates are largely affected by geographic locale, namely the state or region, where a person resides [17].

The estimation of cancer incidence sometimes present difficulties and complexities due to the unavailability of accurate population size data to serve as the denominator for cancer diagnosed in various settings. As a result of the limitation in population data, there are very few studies that have estimated incidence or cumulative incidence in childhood cancer. The current study was proposed due to the lack of data on cumulative incidence of childhood cancer in the state of Delaware, with incidence rate for cancer at all sites of 509 per 100,000 persons (2007–2011); which is higher than the U.S. incidence rate of 460 per 100,000 persons [18]. The recent cancer statistics by the state combined individuals between the ages of 0 through 39 together as one single group, resulting in inability to estimate and compare the pediatric cancer trends in the state [18]. Since we provide cancer care to an estimated >80% of children with cancer in the state, we aimed to examine the distribution of overall childhood malignancies diagnosed in our healthcare system with respect to descriptive cancer epidemiology indicators, namely race, geographic locale, sex, and age at tumor diagnosis.

## 2. Experimental Section

The study was approved by our Institutional Review Board to examine the distribution of cancer in children 0 to 17 years residing in the state of Delaware at the time of their diagnosis, 2004 to 2014. We estimated the overall pediatric cancer cumulative incidence between 2004 and 2014 and stratified childhood malignancies by health disparities indicators, zip code, and county to examine trends and patterns.

### 2.1. Study Area and Population

We collected data on 592 patients from the Nemours Electronic Medical Records (NEMR) diagnosed with pediatric cancer. Of these 592 study subjects, five were excluded because their zip codes did not have 2010 U.S. Census population size data. There were 106 subjects from the remaining 587 who were diagnosed with pediatric cancer in the years 2001 to 2003 and were therefore excluded from the study sample. This exclusion was due to the lack of information on some of the main health disparities indicators examined in this study. Therefore our study population consisted of 481 children, 0 to 17 years old diagnosed with cancer from 2004 to 2014, and followed for the disease. The children population size was obtained from the U.S. Census data, 2010.

### 2.2. Data Source

We used the NEMR which includes data on all types of childhood malignancies from A.I. duPont Children’s Hospital, Wolfson Children’s Hospital in Jacksonville FL, Nemours Children’s Hospital in Orlando, FL, and The Children’s Hospital at Sacred Heart in Pensacola, FL. The Nemours database gathers information on age at diagnosis, height and weight, tumor histology, radiation therapy, chemotherapy, stem cell and bone marrow transplants, exposure to tobacco and smoke, surgery received, family history of cancer, and insurance type. Since cancer exposure data collection in our institution began in 2010, we were unable to assess the effect of tobacco and smoke as well as family history of cancer on healthography. Demographic information including, zip code, sex, race, and ethnicity were available. Population of children under the age of 18 and the median income were extracted for each zip code using the United States Census Bureau American FactFinder based on the 2010 United States Census [19]. For the verification of the population size data and for projected population size variation between 2010 and 2014, we contacted the U.S. Census Office during the period of these data processing.

### 2.3. Variables

This study examined the cumulative incidence of childhood cancer in the NEMR and differences in cancer frequency by race, sex, age at diagnosis, and geographic locale. Race, which was self- reported, was classified as Black, White, or Other. We used this category since granular race and ethnicity data collection in our institution began in 2010. Age at diagnosis was grouped into (a) 0–4 years; (b) 5–9 years; (c) 10–14 years; (d) 15–17 years. Geographic locale was sub-classified by zip codes and counties in Delaware.

### 2.4. Statistical Analysis

We performed summary statistics on variables of interest including zip codes, counties, sex, race, age group, ethnicity, major malignancies, and survival status. To examine the tumor and specific tumor proportion within zip code or race category for instance, we utilized the *proportionate morbidity* estimate, *implying for example, a given race count as numerator divided by the total childhood cancer count as the denominator*. Descriptive statistics were used to examine the percentages of childhood cancer per zip code with the total tumor frequency in the state of Delaware as the denominator. Tumor histology was examined to determine the eight most commonly diagnosed pediatric tumors in our institution. Age-category was used to represent the distribution and percentage of tumors in each age group. Temporal trends were examined by assessing summary statistics on childhood cancer count and percentages for 2004 to 2008 *vs.* 2009 to 2014.

The overall cumulative incidence was estimated by the total number of cancer cases (*n* = 481) divided by the total population of children 0 to 17 years old in Delaware (*n* = 205,765). Cumulative incidence for the eight major childhood malignancies was estimated by the frequency of a specific major tumor divided by the total children population in Delaware. Similarly, cumulative incidence by sex was determined by the cancer count for boys or girls for a specific tumor divided the specific sex population size of children 0 to 17 years old. All estimates for cumulative incidence utilized 100,000 children as the multiplier and age adjusted to the 2010 U.S. standard population.

Whereas the purpose of this study remains descriptive epidemiology of childhood cancer in the state of Delaware with a representative sample form our institution, to examine significant difference between the state as referent and other zip code, we applied inferential statistics to quantify the random error or precision of the observed risk ratio. The tumor diagnosis as count, effect size (10% difference) as well as some zip code population sizes, was too small for sufficient power to detect a statistically significant difference at 0.05 type I error tolerance level. Therefore in this study, the probability value *p* > 0.05 does not necessarily imply “no difference” in comparison to the referent but reflects the small sample size. The Poisson regression model was used to determine the incidence risk ratio for each zip code by using the state of Delaware as the referent, while Pearson chi-squared test was performed to assess the association between major pediatric malignancies and race as well as sex. Due however to the small study size, and the small tumor counts by sex, age group and race indicators, this analysis did not focus on the significant level but on the observed percent difference in the distribution of specific tumor counts by race, age group at tumor onset, and sex.

The geo-mapping and racial mapping of the overall pediatric malignancies was performed by distributing the tumor counts by zip code and by race. In effect given a zip code, tumor counts were populated in that geographic locale and the same applied to race (black, white and other) by geography.

All test were two-tailed and the significance level (type I error tolerance) was set at 0.05 (Poisson regression model). STATA 13.0 (STATA Corporation, College Station, TX, USA) was used for the entire analyses [20].

## 3. Results and Discussion

These data represent cumulative incidence of childhood cancer in the state of Delaware between 2004 and 2014, implying a decade experience with the leading cause of disease- related death among children 0 to 14 years. A total of 481 childhood cancers were diagnosed during this period. The population of children ages 0 to 17 in Delaware was 205,765 children. The cumulative incidence (CI) of childhood cancer was 234 per 100,000 children, while the annual cumulative incidence was 23 per 100,000 children.

### 3.1. Cancer Description by Geographic Locale

**Figure 1:**
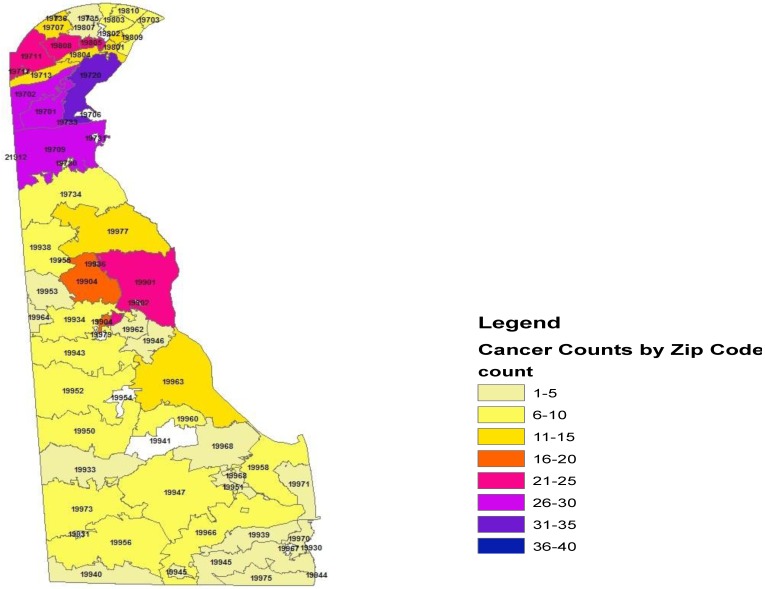
Geo-mapping of the State of Delaware Childhood Cancer, 2004–2014.

Within the state of Delaware, the proportionate cancer morbidity (tumor counts per zip code) indicated the distribution by zip codes: 19701 (*n* = 26, 5.4%), 19702 (*n* = 30, 6.2%), 19709 (*n* = 28, 5.8%), 19720 (*n* = 35, 7.2%), 19805 (*n* = 23, 4.7%), 19808 (*n* = 25, 5.1%), 19901 (*n* = 21, 4.3%), and 19904 (*n* = 20, 4.2%). Figure 1 demonstrates the distribution of pediatric cancer throughout the state of Delaware.

Table 1 illustrates the distribution of cancers as frequencies (proportionate morbidity) in the three counties within the state of Delaware. The cancer counts in New Castle *n* = 296 (65%), Kent County *n* = 91 (18.9%), and Sussex County *n* = 94 (19.5%). These data reflect the proportionate morbidity of cancer within these geographic areas. The cumulative incidence was comparable among the three counties during this time period, New Castle County (*n* = 296, population size = 125,079, CI = 237 per 100,000 children), Kent County (*n* = 91, population size = 40,379, CI = 225 per 100,000), and Sussex County (*n* = 94, population size = 40,307, CI = 233 per 100,000).

**Table 1:** Proportion of pediatric malignancies in the three counties of Delaware relative to the state of Delaware.

County	Cancer Count	Proportion	Standard Error
New Castle	296	0.6515	0.022
Kent County	91	0.1891	0.0179
Sussex	94	0.195	0.0181

**Figure 2:**
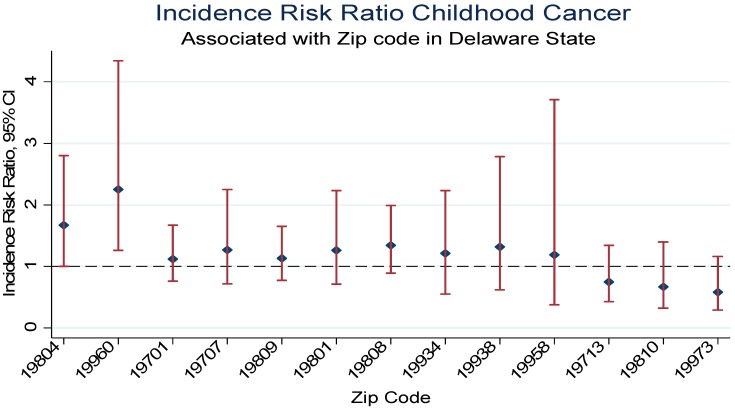
Pediatric Cancer Incidence Risk Ratio Associated with Selective Delaware Zip Codes, 2004–2014.

Table 2 demonstrates the incidence risk ratio of pediatric malignancy by zip code using the state of Delaware as the reference group and the 2010 Census data as the population size for the pediatric age (0 to 17 years). Relative to the state of Delaware the cumulative incidence rate was higher in zip code 19804 with a significant 68% increase risk of developing pediatric cancer of all sites IRR = 1.68, 95% Confidence 1nterval (CI), 1.0–2.8, *p* = 0.04. There were a total of 15 cancers diagnosed with seven malignancies between 2004 and 2008 and eight malignancies between 2009 and 2014 in zip code 19804. Additionally, an estimated nine malignancies were diagnosed among boys and six among girls. Acute lymphocytic leukemia accounted for three cases, CNS/Brain two cases, and lymphoma two cases. Other malignancies diagnosed included Acute myeloid leukemia (AML), renal, Hodgkin’s, endocrine gland, salivary gland, skin, placenta, and cancer of no specific site. Similarly, relative to the state of Delaware children in zip code 19960 were two times as likely to be diagnosed with childhood malignancy (IRR = 2.25, 95% CI, 1.26–4.34). Although imprecise, there were increased risk of childhood malignancies in 19701 (IRR 1.12), 19707 (IRR = 1.27), 19709 (IRR = 1.13), 19801 (IRR = 1.26), 19808 (IRR = 1.34), 19934 (IRR = 1.21), 19938 (IRR = 1.32), 19958 (IRR = 1.19) relative to the state of Delaware. In contrast, childhood malignancies were lower in zip codes 19713 (IRR = 0.75), 19810 (IRR = 0.67), 19966 (IRR = 0.67), 19973 (IRR = 0.58) compared to the state of Delaware. Figure 2 demonstrates the incidence risk ratio for selected zip codes.

**Table 2:** Incidence risk ratio of pediatric cancer by zip code.

Zip Code	Cancer (n)	Population Size	IRR	95% CI
19701	26	9885	1.12	0.76–1.67
19702	30	13732	0.93	0.65–1.35
19703	8	3416	1.00	0.50–2.01
19707	12	4033	1.27	0.72–2.25
19709	28	10628	1.13	0.77–1.65
19711	22	9131	1.03	0.67–1.58
19713	12	6806	0.75	0.43–1.34
19720	35	14748	1.01	0.72–1.43
19730	1	106	4.04	0.57–28.7
19734	6	3275	0.78	0.35–1.75
19801	12	4066	1.26	0.71–2.23
19802	14	6290	0.95	0.56–1.62
19803	9	4482	0.86	0.44–1.66
19804	15	3827	1.68	1.00–2.80
19805	23	10989	0.89	0.59–1.36
19807	3	1614	0.79	0.26–2.47
19808	25	7994	1.34	0.89–1.99
19809	7	2943	1.02	0.48–2.15
19810	8	5117	0.67	0.33–1.34
19901	21	8618	1.04	0.67–1.61
19904	20	8061	1.06	0.68–1.66
19933	5	2201	0.97	0.40–2.34
19934	8	3084	1.21	0.55–2.23
19938	7	2271	1.32	0.62–2.78
19939	3	1075	1.19	0.38–3.71
19940	1	1395	0.31	0.04–2.18
19943	6	2671	0.96	0.43–2.14
19945	3	1376	0.93	0.30–2.90
19946	1	1132	0.38	0.05–2.69
19947	8	4440	0.77	0.38–1.55
19950	7	1603	1.87	0.89–3.94
19951	2	334	2.56	0.64–10.3
19952	7	2557	1.17	0.55–2.47
19953	2	1097	0.78	0.19–3.13
19955	1	13	32.9	4.63–234
19956	10	3992	1.07	0.57–2.00
19958	8	2866	1.19	0.59–2.40
19960	9	1714	2.25	1.16–4.34
19962	5	2923	0.73	0.30–1.77
19963	11	4477	1.05	0.58–1.91
19964	1	325	1.31	0.18–9.36
19966	7	4497	0.67	0.32–1.40
19968	3	2017	0.64	0.20–1.98
19970	4	769	2.22	0.83–5.95
19971	3	1491	0.86	0.28–2.68
19973	8	5942	0.58	0.29–1.16
19975	2	1382	0.62	0.15–2.48
19977	12	5352	0.96	0.54–1.70

**Abbreviations and Notes:** IRR (the cumulative incidence risk ratio); State of Delaware used as the referent for the Poisson Regression. The precision level was 95% Confidence Interval (CI), with the inclusion of 1 between lower and upper levels implying imprecise finding.

Table 3 presents the incidence risk ratio (*Poisson Regression Model*) of pediatric malignancy by county using the state of Delaware as the reference group and the 2010 Census data as the population size for the children age (0–17 years). The cumulative cancer incidence risk was comparable for the three counties albeit a 1% imprecise increased risk in New Castle County during the study period (IRR = 0.01, 95% CI 0.88–1.17).

**Table 3:** Cummulative incidence risk ratio of pediatric cancer by county in DE, 2004–2014.

County	Cancer (n)	Population Size	IRR	95% CI
New Castle	296	125079	1.012	0.88–1.17
Kent	91	40379	0.964	0.77–1.20
Sussex	94	40307	0.997	0.80–1.24

Abbreviation and notes: IRR (the cumulative incidence risk ratio); State of Delaware used as the referent for the Poisson Regression.

### 3.2. Cancer Distribution by Sex and Age at Diagnosis

Table 4 illustrates the cumulative incidence of the eight most commonly diagnosed pediatric malignancies in our sample by sex and anatomic site. Although not shown in the table, cancer counts were slightly higher in boys (52%). The cumulative incidence of ALL was 43.2 per 100,000, CNS/Brain 41.3 per 100,000, renal 16.5 per 100,000, endocrine gland 15.1 per 100,000, lymphoma 15.1 per 100,000, osteosarcoma 14.1 per 100,000, AML 13.6 per 100,000, and connective tissue 13.1 per 100,000. The cumulative incidence varied slightly by sex with boys more likely to be diagnosed with ALL 47.7 *vs.* 38.6 per 100,000 children. Similarly, lymphoma (16.2 *vs.* 13.9 per 100,000) was more common among boys, as well as AML (18.1 *vs.* 8.9 per 100,000). In contrast, girls were more likely to be diagnosed with renal (18.8 *vs.* 14.3 per 100,000) as well as endocrine gland malignancies (18.8 *vs.* 11.4).

Table 5 shows the distribution of pediatric cancer at age at diagnosis. The proportionate morbidity indicates age 0–4 with the most diagnosed cancer (*n* = 153, 32%), followed by age group 10–14 (*n* = 130, 27%).

**Table 4:** The cumulative incidence of the most common pediatric malignancies in Delaware 2004–2014.

Malignancy	Overall Cumulative Incidence per 100,000 Children 2004–2014	Population Subgroup (Sex)	Population Subgroup Cumulative Incidence per 100,000 Children 2004–2014
All Sites	234	Female	230
		Male	237
ALL	43.2	Female	38.6
		Male	47.7
CNS/Brain	41.3	Female	39.6
		Male	42.9
Renal	16.5	Female	18.8
		Male	14.3
Endocrine Gland	15.1	Female	18.8
		Male	11.4
Lymphoma	15.1	Female	13.9
		Male	16.2
Osteosarcoma	14.1	Female	13.9
		Male	14.3
AML	13.6	Female	8.9
		Male	18.1
Connective Tissue	13.1	Female	11.9
		Male	14.3

Abbreviations: ALL: Acute lymphoblastic leukemia; CNS: Central Nervous System; AML: Acute myeloid leukemia.

**Table 5:** Distribution of pediatric cancer by age at diagnosis.

Age Group (Years)	Cancer Count (n)	Percentage (%)
0–4	153	32
5–9	107	22
10–14	130	27
15–17	91	19

### 3.3. Racial Distribution of Pediatric Cancer

**Figure 3:**
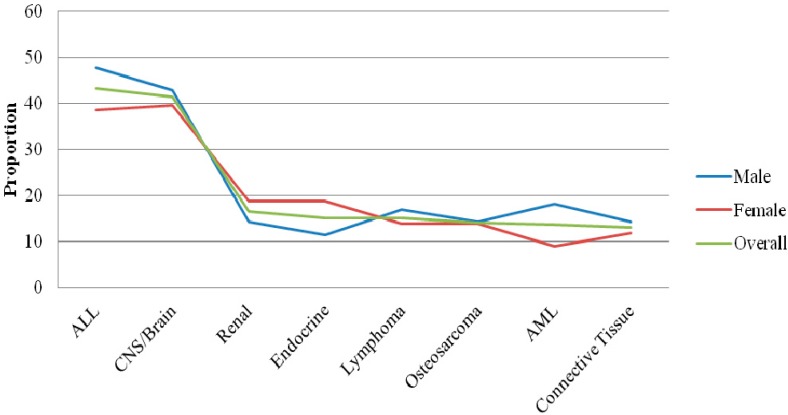
Cumulative Incidence for Childhood Cancer by Sex, Delaware 2004–2014.

**Figure 4:**
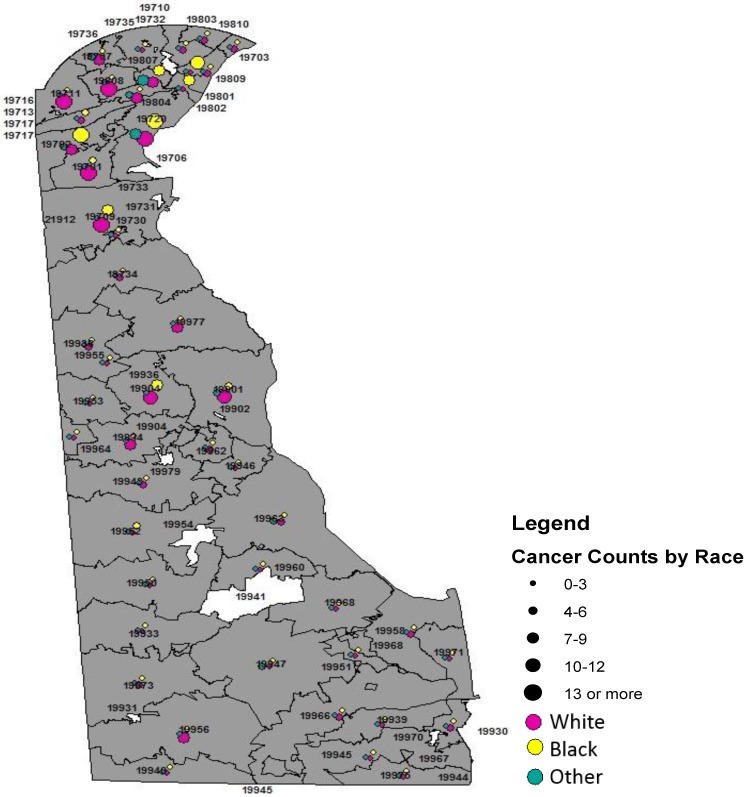
Racial Mapping of Childhood Cancer in Delaware State, 2004–2014.

Table 6 and Figure 3 present the distribution of major childhood malignancies in Delaware by race. Although not shown on the table, more than 56% percent of the tumors diagnosed were in white children, *n* = 268 (56%), blacks, *n* = 120 (25.3%), and other, *n* = 87 (18.3%). White children (210 per 100,000) had a lower cumulative incidence than black children (223 per 100,000). Racial variance was observed in ALL, with whites (20.5%) more commonly diagnosed relative to blacks (10.0%), χ^2^ (2) = 8.3, *p* = 0.02. Although imprecise or statistically marginally significant, CNS/Brain tumors were more commonly diagnosed among blacks (20.8%) relative to whites (16.8%), χ^2^ (2) = 1.0, *p* = 0.62. Similarly although imprecise, renal carcinoma was more commonly diagnosed among blacks (10.0%) compared to whites (5.6%) χ^2^ (2) = 2.7, *p* = 0.36. Likewise, although imprecise, blacks (8.3%) were slightly more likely to be diagnosed with endocrine gland tumors relative to their white (6.3%) counterparts, χ^2^ (2) = 1.19, *p* = 0.55. Among whites endocrine gland malignancy was more common among girls (70.6% *vs.* 29.4%) but was equally distributed among blacks (50.0% *vs.* 50.0%). Figure 4 shows the racial distribution of pediatric cancer by Delaware zip code.

**Table 6:** Major childhood cancer distribution by race in Delaware 2004–2014.

Malignancy	White n (%)	Black n (%)	Other n (%)	χ^2^ (df)	*p*
ALL	55 (20.52)	12 (10.00)	21 (24.14)	8.30 (2)	0.016
CNS/Brain	45 (16.79)	25 (20.83)	15 (17.24)	0.953 (2)	0.621
Renal	15 (5.60)	12 (10.00)	5 (5.75)	2.72 (2)	0.256
Endocrine Gland	17 (6.34)	10 (8.33)	4 (4.60)	1.19 (2)	0.552
Lymphoma	18 (6.72)	5 (4.17)	7 (8.05)	1.45 (2)	0.484
Osteosarcoma	17 (6.34)	9 (7.50)	3 (3.45)	1.50 (2)	0.471
AML	14 (5.22)	9 (7.5)	5 (5.75)	0.778 (2)	0.678
Connective Tissue	15 (5.60)	7 (5.83)	5 (5.75)	0.0094 (2)	0.995

Notes and abbreviations: Percentages do not sum to 100% since we only examined the 8 most diagnosed malignancies; ALL: Acute lymphoblastic leukemia; CNS: Central Nervous System; AML: Acute myeloid leukemia; χ^2^ (df): chi-square statistic, degrees freedom; *p*: probability value.

### 3.4. Temporal Trends

Table 7 illustrates temporal trends in pediatric cancer in our sample. Using the time trend 2004–2008 and 2009–2014 we observed a comparable distribution of tumor counts by these time periods. Despite the imprecise population sizes, more tumors were diagnosed during the first period (2004–2008).

**Table 7:** Temporal Trends and Cancer Count (2004–2008) and (2009–2014).

Time Period	Cancer Count (n)	Percentage of Cancer (%)
2004–2014	481	100
2004–2008	260	54
2009–2014	221	46

### 3.5. Discussion

The basis for analytic cancer epidemiology is the descriptive finding, which allows for hypotheses generation in order to examine cancer determinants, risk markers, progression, and survival. With the limitations in this area (descriptive childhood cancer epidemiologic data) in the state of Delaware, we proposed to characterize childhood malignancies by geographic locale (zip code), sex, race, as well as temporal trends, and age at onset. There are a few relevant findings from our study. First, the cumulative incidence (period prevalence) was 234 per 100,000 children, implying an annual cumulative incidence of 23 per 100,000 children. Secondly, childhood cancer varies by geographic locale namely zip code. Thirdly, ALL significantly varied by race and brain/CNS was statistically marginally significant, while renal, endocrine gland, AML, lymphoma, and osteosarcoma although imprecise varied by sex and age at onset.

Cumulative incidence of cancer remains an important finding in understanding the burden of malignancies across specified populations. We have demonstrated that the cumulative incidence (period prevalence) of childhood cancer in the state of Delaware is 234 per 100,000 children, implying an annual cumulative incidence of 23 per 100,000 children. Our findings indicate an increase relative to the National Cancer Institute (NCI) data on the annual incidence for childhood cancer in the nation [21]. The observed increase in annual cumulative incidence in DE may be due to several individual and ecologic factors including though not limited to the environment, parental lifestyle and occupation, exposure such as passive and first-hand smoking, socio-economic status as well as exposure of children to ionizing radiation [16]. In contrast, this increase may be due to changes or differences in the classification of childhood malignancy and the age categories used to define childhood tumors. We used the ICD-9 classification code, implying cancer registries or systems that use different classification code may have different pediatric tumor prevalence. We were unable to compare the observed cumulative incidence with the Delaware Cancer Registry which is maintained by the Delaware Health and Social Services (DHSS) Division of Public Health, since the DHSS presented their recent findings on age- specific all site cancer incidence in the years 2007 to 2011 with an overlapping interval of 0 to 39 years of age (incidence rate = 54.6 per 100,000 population) for age at diagnosis [18].

We observed significant geographic variation in our data using geo-coding mapping based on the zip codes in the state of Delaware. The zip codes with highest cumulative incidence were 19804 (Newport) and 19960 (Lincoln). The observed cumulative incidence in these zip codes is indicative of some environmental factors or other known or unmeasured potential carcinogens as well as cancer predisposing factors including, though not limited to, lifestyle variables, paternal occupation, pre and postnatal exposure, maternal hormone, host factor (Fanconi’s anemia, ataxia-telangiectasia, Down syndrome, neurofibromatosis), family history or gene-environment interaction [16]. Risk and predisposing factors assessment in childhood cancer remains complex and often inconclusive, given the small size of these studies, and the lack of rigorous examination of records which tends to limit the inference drawn from these studies. Additionally some studies do not assess or control for confounding, resulting in biased findings leading to inconsistent associations (none, positive, negative).

While cancer has a very long induction period and insulting pathogens may never be known [22], the presence of potential carcinogens in a physical environment may predispose to cancers hence, evidence of excessive cumulative incidence in some geographic locale. Whereas environmental data were not available to us at the time of this study, it is plausible to expect these zip codes to be affiliated with industrial areas or zones in the state of Delaware that may have a relatively excess poor air quality and other environmental toxins. Parental occupation mainly hydrocarbon-related work, metal-related work and radiation-related work had been implicated in childhood cancer development. Some studies have shown association between lead and Wilms tumors, as well as acute non-lymphocytic leukemia, metals and hepatocellular carcinoma, and metal-related work and brain tumor [16]. The zip code 19804 in Newport is affiliated with chemical companies as well as automotive industries. Some epidemiologic data associate hydrocarbon- related work namely service station attendants, motor vehicle mechanics, machinists, lumberman, painters, dyers and cleaners with childhood cancer [23], while paternal occupation mainly gas station attendants, automobile or truck repair, and aircraft maintenance is linked with infant leukemia, and maternal occupation mainly laundry/dry cleaning has been related with Wilm’s tumor [24]. Brain and CNS tumors in children has been associated with parental occupation namely electricians, electrical and electronic workers [16,23]. The companies in this zip code, and the products generated have been implicated as potential carcinogens [25,26,27,28]. In effect, this may very well explain why childhood cancer cumulative incidence was excessive in Newport compared to the state of Delaware cumulative incidence. While the timing of exposure around preconception, and gestation is important in the implication of these potential carcinogens in childhood cancer, it is relevant to note the difficulties in conclusive inference, given several limitations of these often case-control studies, namely recall bias, broad categories of employment, information abstraction from administrative data not intended for research purpose, and multiple agents exposure. Notwithstanding the uncertainties in the implications these parental occupation related carcinogens, our findings of excess pediatric cancer in this zip code support other data associating this geographic locale with all age highest cancer incidence in the state of Delaware [28].

Lincoln, zip code 19960, is a largely agriculturally-based community, indicating the possibility for potential carcinogens around the environment, given the nature of pesticides used on the land that may affect the respiratory tract as well as other organ systems involving inhalation or ingestion [27]. Paternal, gestational and postnatal exposure to pesticides among agricultural workers had been associated with childhood cancer [16,23]. This geographic locale is associated with a large area of farmland, implying the potential use of parental pesticide as well as external exposure on clothing to drive the excessive childhood cancer incidence in Lincoln. While pesticide exposure has been implicated in cancer carcinogenesis, the specific mechanism with a given pesticide has not been fully understood, however the pathway to carcinogenesis involves bioaccumulation and the potential damage to the DNA, inhibiting apoptosis (programmed cell death) [27,29].

Additionally, because of the genetic predisposition to cancer, it is not unlikely to expect the populations in these zip codes with the highest cumulative incidence of pediatric cancer to have a family history of cancer relatively higher than other zip codes, indicative of epi-genetic predisposition. Despite this possibility, it is highly unlikely that the excessive cumulative incidence of childhood cancer in these zip codes (19804 and 19960) is due to genetic, family history of cancer or host factors only, but gene-environment interaction remains a possible explanation. Overall, our findings support previously published data on cancer incidence in both adult and childhood population by geographic locale in Delaware [26].

Black children had a higher cumulative incidence of cancer of all sites than whites (223 per 100,000 *vs.* 210 per 100,000). Epidemiologic data continue to associate higher incidence of childhood cancer, ALL, and thyroid with white children [6,7], however our data did not support this observation with overall childhood cancer. Acute lymphoblastic leukemia (ALL) was more common among white children, affirming previous data [7,8]. Studies on social class had shown the link between higher socio-economic status (SES) and increased risk of leukemia [16], and the increased risk of ALL among whites may reflect race as SES surrogate. Data are lacking on overall childhood cancer incidence and SES. However it is worthy to note that these studies may be difficult to interpret without data completeness for the assessment of confounding and effect measure modifier prior to the inference drawn. The current study supports previous finding by Holmes *et al.* on leukemia using SEER data from the 13 registries, 1973–2006 [8], as well as recent findings that clearly illustrated the incidence disadvantage of whites [7]. Renal carcinoma was more common among blacks relative to whites in our sample. This observation is comparable to other data sources and previous studies in this direction [7].

This study observed sex variability in renal carcinoma, thus affirming previous studies comparing the incidence of renal carcinoma between boys and girls [7]. Furthermore, our data supports biologic variation in female urethra and the potentials for increasing anatomic insult compared to male urethra. While infection with in utero viral pathogens mainly influenza in childhood leukemia, herpesviruses with childhood cancer, as well as Wilm’s tumor with mothers diagnosed vaginal infections [16], these data remain very inconclusive. Additionally, endocrine gland malignancy was more common among white girls relative to black girls and white boys. Endocrine tumors tend to be more prevalent in women compared to men [9]. This observation has been explained by the predominant role of estrogen in endocrine tumors in women. While specific data are not available to our knowledge, it is plausible that the increased prevalence of endocrine tumors in girls is associated with hormonal involvement and imbalance between estrogen and progesterone [9]. AML as well as lymphoma was more common in boys relative to girls. AML, which is relatively more common among boys being a very aggressive malignancy, may explain why boys have survival disadvantage following overall childhood cancer diagnosis and specifically leukemias. However, there is no clear and specific biologic mechanism to account for the preferential occurrence of this malignancy in boys.

Although difficult to establish, we observed temporal trends or patterns in pediatric cancer in our sample. The observation of increasing trend in 2004–2008 may be due to the spike in tumor cumulative incidence in 2004. A possible explanation for the spike was that local private oncology practices paired with A.I. duPont Hospital for Children, implying increased patient volume.

Despite the strength of this descriptive clinical epidemiology of childhood cancer in the state of Delaware, there are some limitations. First, the population sizes for the overall pediatric population in the state as well as the zip codes vary from year to year. In effect, using the Census population size for 2010 may indicate an underestimation of the children population size. However, this underestimation because it is a non-differential misclassification may not affect the cumulative incidence risk ratio of childhood cancer by geographic locale (zip code) if the population projection is comparable across the zip codes. Secondly, our inability to compare these findings with that of the state of Delaware Health and Social Services as a result of the state’s cancer registry recent findings that lump pediatric cancer with adult malignancy by using the age at diagnosis category of 0 to 39 years. Thirdly, Nemours Data Warehouse does not communicate in terms of record linkage with the Delaware Cancer Registry, which may result in the possibility of tumor diagnosis misrepresentation. Fourthly, due to the lack of individual level income data, we were unable to characterize tumor distribution by income level, restricting our ability to examine the effect of SES on childhood cancer incidence in our sample. Lastly, our descriptive cancer estimates may indicate underestimation due to children from the state of Delaware receiving cancer treatment from other hospitals, namely, the Children’s Hospital of Philadelphia (CHOP). Despite this flux to other states for care, our cancer patients represent an estimated 80+% of children with cancer in the state of Delaware, implying a representative sample for a reliable estimate.

## 4. Conclusions

In summary, the cumulative incidence of childhood cancer as well as the annual cumulative incidence is higher in the state of Delaware relative to the nation. There are geographic variation in all-sites cancer, sex variations in renal carcinoma and endocrine tumor, while racial variability in leukemia remains.
